# Child temperament as a longitudinal predictor of mother–adolescent interaction quality: are effects independent of child and maternal mental health?

**DOI:** 10.1007/s00787-023-02359-6

**Published:** 2024-01-19

**Authors:** Leonie Fleck, Anna Fuchs, Katharina Williams, Eva Moehler, Franz Resch, Julian Koenig, Michael Kaess

**Affiliations:** 1 Department of Child and Adolescent Psychiatry, Centre for Psychosocial Medicine, University of Heidelberg, Heidelberg, Germany; 2 Department of Child and Adolescent Psychiatry, Saarland University Medical Center, Homburg, Germany; 3 Department of Child and Adolescent Psychiatry, Psychosomatics and Psychotherapy, Faculty of Medicine and University Hospital Cologne, University of Cologne, Cologne, Germany; 4 University Hospital of Child and Adolescent Psychiatry and Psychotherapy, University of Bern, Bern, Switzerland; 5 Institute of Psychology, University of Heidelberg, Heidelberg, Germany

**Keywords:** Temperament, Mental health, Parent–child interaction, Adolescents

## Abstract

Adaptive parent–child interaction plays a major role in healthy child development. Caregiver mental health problems can negatively impact parent–child interaction. In turn, interactional quality is often studied as a predictor of child outcome. However, child characteristics supposedly shape parent–child interactions as well. Given associations between child and caregiver mental health and child temperament, this study aimed at differentiating their effects on dyadic interaction quality in adolescence. Child temperament and character at age 5 were investigated as longitudinal predictors of observed mother–adolescent interactional quality at age 14 in a community sample (*N* = 76). It was examined whether these effects were independent of maternal and child mental health and earlier dysfunctional interaction. Lower novelty seeking, higher reward dependence, and higher cooperativeness separately predicted higher dyadic interactional quality at age 14. Controlling regressions for dysfunctional interaction at age 5, which was a significant negative predictor of later interactional quality, cancelled out the effects of novelty seeking and cooperativeness. Past or concurrent maternal or child psychopathology did not explain variance in mother–adolescent interaction. Applying backward selection, a model including reward dependence and dysfunctional interaction at age 5 and concurrent maternal stress showed the best fit for explaining dyadic interaction quality. Results suggest that enduring rather than transient child features predict interactional quality in a community sample. Effects of temperament are not better explained by those of psychopathology, but a combination of child, maternal, and dyadic features predicted dyadic behaviour best. Selective prevention should target parenting in the context of challenging child characteristics specifically.

**Supplementary Information:**

The online version contains supplementary material available at 10.1007/s00787-023-02359-6.

## Introduction

### Meaning of adaptive caregiver–child interaction

Caregiver–child interactions and their adaptiveness play a major role in healthy child development [1]. Mutuality, reciprocity, emotional availability, and sensitivity are terms describing the nature of caregiver–child interactions that foster secure attachment and mental health. Caregiver psychopathology is a factor often found to negatively impact interaction quality [2–4]. The finding that maladaptive caregiver–child interaction is in turn predictive of child psychopathology [5] infers a causal relationship and a possible path for intergenerational transmission. Synchronous or reciprocal dyadic behaviour, i.e., caregivers and children engaging in mutually adaptive give-and-receive interactions, is a consistent positive predictor of children’s self-regulation abilities [6, 7]. They could thus function as buffers to the development of mental disorders. Yet, as is suggested by the transactional model of development which emphasizes the role of bidirectional interplays between child and environment [8, 9], not only the caregiver but also child characteristics shape the interaction. Consequently, it is important to study not only caregiver but also child characteristics that would *predict interactional quality* in the long term, and to examine whether effects of more enduring characteristics such as temperament can be differentiated from those of psychopathology. Different professionals working with children and/or parents could benefit from being informed about traits which might challenge the development of interactional ability.

### Predictors of caregiver–child interaction

#### Child temperament

Although caregiver–child interaction has often been studied as a predictor of child outcome, the transactional model proposes that child characteristics shape interactions with caregivers as well [8, 9]. Children differ widely in the reactions towards the environment, including their reaction to novel stimuli, frustration, emotionality, and sociability. Individual differences in behavioural and emotional response tendencies are referred to as temperament [10, 11]. As outlined in [12], temperament can have either direct or indirect linear effects on social development: Regarding direct effects, either the temperamental feature is synonymous with the outcome (such as with inhibition and social withdrawal), or a temperamental feature can directly impact an outcome (such as aggressive reactions towards frustrations in a child with high reactivity). An indirect effect can occur when children’s characteristics and behaviours shape reactions of the environment, which in turn influences child development. Naturally, certain traits will pose more challenges on interactions and parenting than others. E.g., in a short-term longitudinal and a cross-sectional study, infant and toddler negative emotionality predicted maternal power assertion, respectively negative affective states of the dyad [13, 14].

Other studies examined bidirectional influences of child temperament and caregiver–child interaction. Transactional theories pose that child and parent characteristics mutually influence each other. Maternal features such as bonding impairment and negativity towards the child predict subsequent child temperament, but also child characteristics predict subsequent maternal behaviour [15–17], although [15] found behavioural inhibition to predict parental structuring but not vice versa. Hence, influences seem to work in both directions and children play a major role in shaping their environment and the caregiver–child relationship. Many studies’ longitudinal designs spanned across several months. Therefore, childhood temperament as a long-term predictor of mother–child interaction passing several developmental periods is of specific interest.

#### Caregiver and child mental health

One factor regularly studied as a predictor of caregiver–child interactions is parent mental health. Maternal depression, borderline personality disorder, and anxiety are among the mental health conditions most studied in relation to mother–child interaction quality. Research has shown that maternal depression negatively impacts mother–child interaction, including lower sensitivity and lower touch and gaze synchrony and more withdrawn behaviour [2, 18, 19]. Anxious mothers showed more intrusive and controlling behaviours and lower facial affective coordination during interaction with their offspring [2, 20, 21]. Maternal borderline personality disorder is usually linked to lower sensitivity and synchrony and more intrusive or hostile behaviours [4, 22, 23]. A link has also been found between parenting stress and lower interactional synchrony [24]. Thus, mother–child interactional quality may be altered in the context of maternal psychopathology and stress. Altered mother–child interaction patterns are one of the important pathways for intergenerational transmission of mental health impairments [5]. However, a link between maternal psychopathology and decreased mother–child interactional quality has not always been confirmed [25]. Consequently, additional factors must explain variance in caregiver–child interaction.

Child mental health has mostly been researched as an outcome of parent–child interactional quality. More negative maternal and dyadic behavioural patterns during interaction have been associated with child behaviour problems, depression, and self-harm [24, 26–29]. According to transactional models, it is also likely that aspects of child mental health problems shape caregiver–child interaction. In sum, dyads with either caregivers or children suffering from mental health problems are at higher risk for maladaptive interaction styles.

#### Associations between child temperament and child and caregiver mental health

Child temperament is often found to correlate with child psychopathology. Behavioural inhibition together with low effortful control correlates with internalizing problems, and impulsiveness or anger-irritability together with low effortful control relate to externalizing symptoms [30]. From a spectrum perspective in developmental psychopathology, this relationship comes to place because psychopathology constitutes an extreme on the same continuum as temperament [31]. An alternative vulnerability perspective poses that there is a certain degree of discontinuation between temperament and psychopathology, but temperamental traits together with other factors make an individual more vulnerable or resilient towards the development of (certain types of) psychopathology. Both temperamental traits and mental health problems could impact mother–child interaction, but given their correlation, their effects might be difficult to distinguish. The question arises whether child temperament will explain mother–child interaction above and beyond the effect of child mental health problems.

There are also associations between parental mental health and child temperament [32–35]. Studies often utilize parental reports of child temperament, which could cause methodological issues when using the same informant for both outcomes. Associations might be either due to heritable vulnerability, i.e., true associations, or due to altered reporting or perception as a function of parental psychopathology, i.e., due to biased reporting, which is a concern of authors in the field [36]. A methodological study, however, found that there was no evidence for biased reporting of child temperament due to maternal concurrent and past psychiatric symptoms [37]. If this leads to the conclusion that associations between child temperament and parental mental health are valid, it is still of interest to determine whether they both independently contribute to dyadic interaction quality.

### Present study

**Fig. 1:**
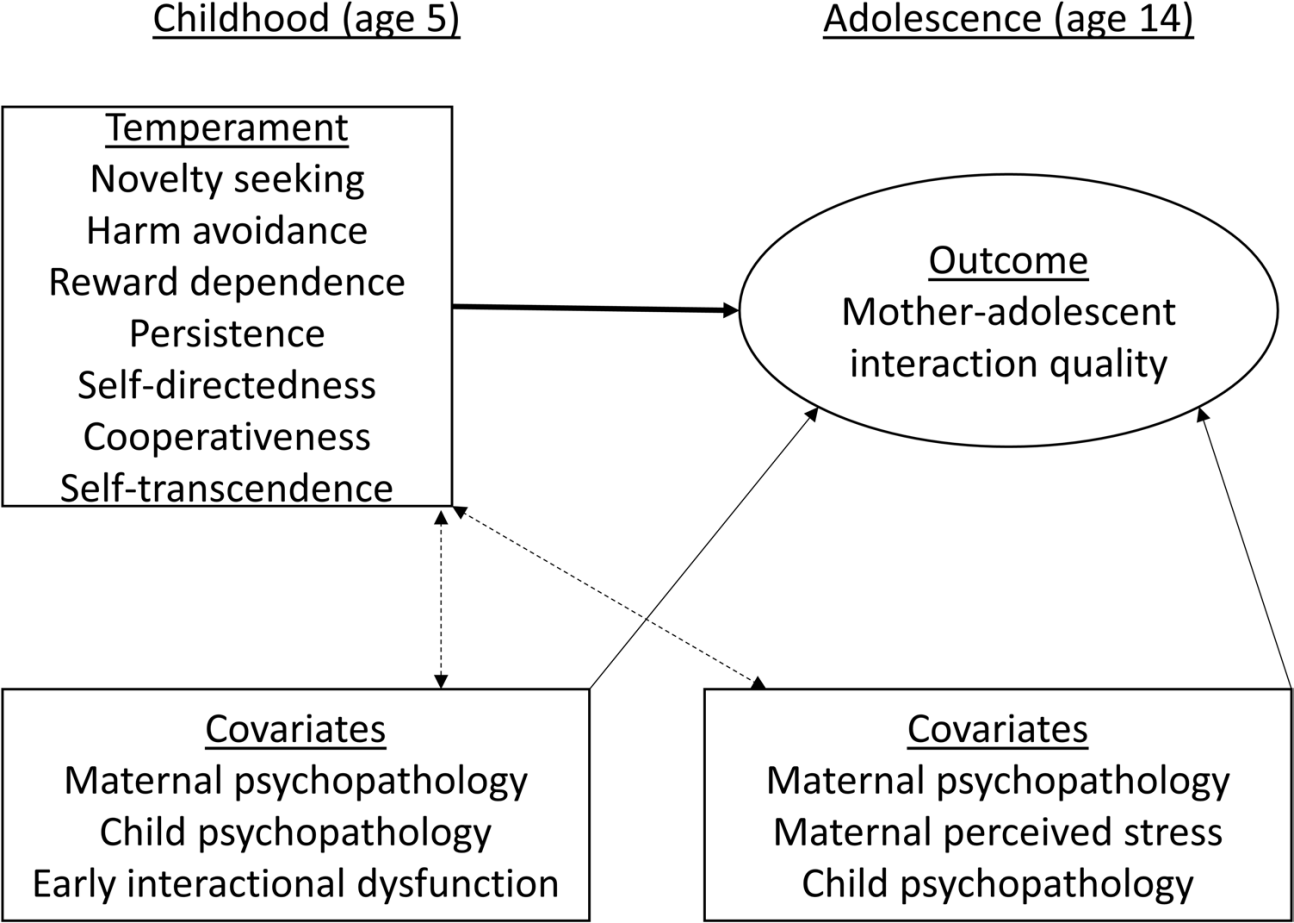
Conceptual figure of the assumed interrelations between temperament, mother–adolescent interaction quality, and covariates (note that the bold solid line represents the main research question of how childhood temperament traits relate to later mother–adolescent interaction quality. Based on the literature, childhood and adolescent mental health covariates and early interactional dysfunction should also predict mother–adolescent interaction quality (solid lines). Dashed lines indicate proposed interrelations between temperament and covariates)

First, we aimed to examine which child temperament and character traits in preschool age would predict mother–adolescent dyadic interaction quality. We assessed temperament based on Cloninger’s psychobiological model of temperament and character [10]. Four temperament dimensions describe biologically based response tendencies. *Novelty seeking* (characterized by impulsivity, exploratory excitability, and disorderliness) and *harm avoidance* (fear, shyness, and fatigability) show correlations with externalizing and internalizing psychopathology in youth, respectively [38]. Children with strong behavioural activation or inhibition might also be more challenging to parent, and difficulties could result in lower dyadic interactional quality. *Persistence* (eagerness of effort, ambition) and *reward dependence* (sentimentality, social attachment, dependence on approval) are supposed to be resources that predict mature character development. Character is described by the dimensions of *self-directedness* (self-acceptance, purposefulness, and resourcefulness), *cooperativeness* (compassion, social acceptance) and *self-transcendence* (spirituality, fantasy). High levels on all character dimensions are supposed to reflect maturity in age-adequate personality development. Hence, they should be a resource in interpersonal interaction. It was thus hypothesized that higher interactional quality would be observed in dyads in which children were *lower* in a) novelty seeking and b) harm avoidance, and *higher* in c) reward dependence, d) persistence, e) self-directedness, f) cooperation, and g) self-transcendence. Links to reward dependence and cooperativeness would be expected in particular, as these dimensions directly relate to interpersonal response and functioning. A composite score of maternal, paternal, and preschool teacher reports of child temperament and character was obtained which reflects the child’s behaviour in various settings. Second, we wanted to explore whether these traits would explain later mother–adolescent interaction quality above and beyond past and concurrent child and maternal mental health problems and past dysfunctional interaction. See Fig. 1 for a conceptual figure.

## Methods

### Procedure

The study was approved by the local ethics committee (Faculty of Medicine at the University of Heidelberg, S-553/2016). In this longitudinal investigation, mothers and their children took part in six assessment time points (t1 = 2 weeks after birth, t2 = 6 weeks, t3 = 4 months, t5 = 5.5 years, and t6 = 14 years) between the years 2002 and 2017. Mothers provided written informed consent for all assessment time points, as did adolescents at t6. The current article comprises data from t5 and t6. At t5, mothers completed questionnaires on their own and the child’s well-being and child temperament. At t6, mothers and adolescents participated in a three-hour appointment, including the completion of questionnaires and two mother–adolescent interaction tasks.

### Participants

Initial recruitment of mothers took place in four major local obstetric units and offices and via newspapers in the area of Heidelberg, Germany, in the years of 2002–2003. Inclusion criteria were full-term deliveries, infant weight > 2500 g, APGAR scores > 7, and good infant health at the first three postnatal examinations. Mothers with insufficient German language comprehension, use of drugs or medication possibly risking fetal health, smoking > 5 cigarettes per day, or consuming alcohol during pregnancy were excluded from participation. Originally, *n* = 102 mothers participated at t1. At t5 and t6, *n* = 88 (86%) and *n* = 76 (75%) families remained in the study, respectively.

### Measures

*Junior Temperament and Character Inventory 3-6R (JTCI 3-6R).* At t5, child temperament and character dimensions were assessed using mother, father, and preschool teacher reports of the JTCI 3-6R, based on Cloninger’s Psychobiological Model of Temperament [10, 39]. The JTCI 3-6R uses a 5-point Likert scale ranging from 0 to 4. The temperament scales describing biological-based response tendencies comprise Novelty Seeking (15 items, α = 0.89), Harm Avoidance (16 items, α = 0.92), Reward Dependence (12 items, α = 0.75), and Persistence (12 items, α = 0.84). The character dimensions include Self-Direction (11 items, α = 0.84), Cooperativeness (10 items, α = 0.87), and Self-Transcendence (10 items, α = 0.77). Character describes differences in intentional goals and self-concepts, which are, according to Cloninger’s model, developed based on propositional learning.

*Parent–Child Dysfunctional Interaction (PCDI).* At t5, the Parenting Stress Index-Short Form was administered [40]. The parent–child dysfunctional interaction subscale (PCDI) (12 items, α = 0.75) was used as a marker of earlier mother–child interactional difficulties. The 5-point Likert scale ranges from 1 (strongly agree) to 5 (strongly disagree).

*Strengths and Difficulties Questionnaire (SDQ).* Mothers and preschool teachers (t5) completed the SDQ assessing children’s emotional and behavioural problems [41]. At t6, the adolescent self-report version was used. The SDQ comprises the five subscales emotional problems, conduct problems, hyperactivity, peer problems, and prosocial behaviour. The total problem score is calculated by summing up the four problem subscales (20 items, t5 α = 0.74; t6 α = 0.81). Items are scored on 3-point Likert scale ranging from 0 (not true) to 2 (certainly true).

*Symptom Checklist 90R (SCL-90R).* At t5, mothers reported on their current psychological distress using the SCL-90R [42]. The questionnaire covers somatization, obsessive–compulsive symptoms, interpersonal sensitivity, depression, anxiety, anger–hostility, phobic-anxiety, paranoid ideation, and psychoticism. The global severity index reflects the overall extant of distress (90 items, α = 0.95). A 5-point Likert scale ranges from 0 (not at all) to 4 (extremely).

*Brief Symptom Inventory-18 (BSI-18).* At t6, mothers responded to the BSI-18 [43], a short 18-item version of the SCL-90R comprising the scales depression, anxiety and somatization. Again, the global severity index reflects overall levels of distress (α = 0.82).

*Perceived Stress Questionnaire (PSQ).* Mothers reported on their subjective stress using the PSQ (t6) [44]. The PSQ covers items about perceived worries, tension, joy, and demands. The subscale “joy” is reversed before calculation of the total score (20 items, α = 0.76). A 4-point Likert scale ranges from 1 (almost never) to 4 (usually).

*Coding Interactive Behaviour (CIB).* At t6, mothers and adolescents engaged in two mother–child interaction paradigms. They were first asked to both pick a fun activity they would like to do with each other and plan these activities for 10 min. Next, they were asked to both pick a topic of conflict relevant to their everyday lives and discuss these for 10 min. Interaction was rated using the CIB [45]. The CIB comprises 56 behavioural codes which are grouped into theoretically meaningful scales describing parental, child, and dyadic behaviour. Dyadic behaviour as the target of the current investigation comprises the scales dyadic reciprocity (reciprocity, compatibility, fluency, α = 0.88) and dyadic negativity (tension, constriction, α = 0.77), i.e., a total of 5 items which can be rated on a scale from 1 (indicating absence or very low quality of a behaviour) to 5 (indicating high presence or quality of that behaviour). Rather than specific behaviours, the dyadic scale describes the extent to which caregiver and child engage in a mutually adaptive and satisfying give-and-receive interaction, and evaluates the global emotional “temperature”. CIB dyadic behaviour, therefore, displays the degree of behavioural reciprocity in a dyad. Twenty-four dyads were coded by more than one rater, with an interrater-reliability of 88% (κ = 0.78). For the purpose of the current study, dyadic negativity during both interactions was subtracted from dyadic reciprocity during both interactions, creating a “dyadic interaction quality” variable with higher scores indicating a higher degree of behavioural reciprocity across both interaction paradigms.

### Data analysis

All analyses were carried out using Stata 16 (College Station, TX: Stata-Corp LLC). Prior to the analyses, mothers’, fathers’, and preschool teachers’ JTCI reports as well as mothers’ and preschool teachers’ t5 SDQ reports were averaged, resulting in one mean score per scale. Possible differences were examined between families who dropped out of the study and those who retained. Independent sample Welch’s *t *tests were used to analyse differences regarding continuous variables. χ^2^ tests were used for categorical variables. The significance level for all analyses was set to *p* < 0.05.

For better interpretation, all predictors were z-standardized prior to analysis. Both research questions were approached using both a backward selection procedure and separate investigation of the scales: Regarding research question 1, first, a multiple linear regression analysis was performed to obtain the overall amount of variance in dyadic interaction quality at age 14 explained by all child temperament and character dimensions at age 5. Next, using backward stepwise selection, a model containing those temperament and character scales with the best predictive value was obtained. Alpha for removal of variables from the model was set to *p* > 0.05. Alpha for adding variables to the model was set to *p* ≤ 0.05. Additionally, we performed separate regression analyses for each of the temperament and character dimensions.

Regarding research question 2, we first performed another backward selection process, now including the set of covariates (child emotional and behavioural problems at t5 and at t6, maternal psychopathology at t5 and at t6, maternal perceived stress at t6, and mother–child dysfunctional interaction at t5) in addition to the selected temperament and character scales. With regard to the temperament and character scales which significantly predicted dyadic interaction quality in separate regressions, it was tested whether their effects remained stable after controlling for the specific covariates. This time, covariates were added to those temperament and character dimensions in separate models. If, upon addition of the covariate, the F test of the overall regression model becomes non-significant, their addition does not benefit the model and the respective temperament or character trait is a better single predictor of dyadic interaction quality. Similarly, if the F test stays significant and so does the coefficient of the effects of the temperament or character trait, it suggests that the effect of temperament is at least partially independent of that of the covariate.

*N* = 1 family did not submit any t5 questionnaires and *n* = 1 family missed t5 child mental health and dysfunctional interaction questionnaires (see Table S1). Applying listwise deletion, the final analyses sample comprised either *N* = 75 or *N* = 74 families.

## Results

### Sample description

Children of families who dropped out of the study between t5 and t6 did not differ from those who remained with respect to any of the temperament or character dimensions, maternal psychopathology, child emotional and behavioural problems, maternal partnership, education, or child sex at t5. There was a significant difference regarding mother-reported dysfunctional parent–child interaction, indicating mothers who remained in the study reported more dysfunctional parent–child interaction (*M* = 17.59, *SD* = 0.48) than dropouts (*M* = 15.27, *SD* = 0.59;* t*(29.38) = −3.05, *p* = 0.005). Mothers who remained in the study were significantly older (*M* = 33.76, *SD* = 0.49) than dropouts (*M* = 31.96, *SD* = 0.69;* t*(52.31) = −2.14, *p* = 0.037).

At t6, mothers who retained were *M* = 48.22 years of age. Adolescents were 14.0 years old, and 35 (46.05%) were female. Most adolescents went to grammar school (84.21%), followed by adolescents attending intermediate secondary school (14.47%) and other school types (1.32%). The majority of mothers was in a partnership with the child’s father (82.89%) or a different partner (7.89%), whereas 9.21% held no current partnership. Most mothers held a university degree (69.74%), followed by mothers with intermediate secondary school (19.74%) or a university entrance diploma (10.53%). Supplement Table S1 shows the descriptive statistics of all the study variables in the current sample.

### Research question 1: which childhood temperament and character features longitudinally predict mother–adolescent dyadic interaction quality?

A multiple regression model containing all temperament and character dimensions from the JTCI as predictors of dyadic interaction quality was significant (*R*^2^ = 0.21, *F*(7, 67) = 2.52, *p* = 0.023). However, in this full model, none of the single coefficients reached significance. The backward selection approach determined that a multiple regression model in which reward dependence (*β* = 0.29, *p* = 0.010) and cooperativeness (*β* = 0.24, *p* = 0.031) positively predicted interaction quality, and harm avoidance negatively predicted interaction quality (*β* = −0.24, *p* = 0.028) fit the data best.

In separate regression analyses, higher reward dependence (*β* = 0.31, *p* = 0.006) and higher cooperativeness (*β* = 0.26, *p* = 0.026) at age 5 significantly predicted higher levels of dyadic interaction quality at age 14, reflecting results of the multiple regression model. In contrast, instead of harm avoidance, novelty seeking was a single significant negative predictor of interaction quality (*β* = −0.25, *p* = 0.026). Persistence, self-directedness, and self-transcendence were not significantly associated with later quality of dyadic interaction patterns in either the multiple or separate regression models. See Table 1 for a detailed display of regression results.

**Table 1:** Regression analyses: temperament and character (t5) predicting dyadic interaction quality (t6)

Variables	*β*	*b*	*SE*	*t*	*p*	F	Df	*p(F)*	*R*^*2*^	*Intercept*
*Full regression model*										
Novelty seeking	−0.09	−0.29	0.64	−0.46	0.650	2.52	7, 67	**0.023**	0.21	3.43***
Harm avoidance	−0.17	−0.51	0.55	−0.95	0.348					
Reward dependence	0.26	0.77	0.40	1.92	0.059					
Persistence	−0.06	−0.17	0.45	−0.38	0.707					
Self-directedness	0.09	0.29	0.65	0.45	0.658					
Cooperativeness	0.17	0.51	0.60	0.84	0.404					
Self-transcendence	0.10	0.30	0.40	0.74	0.465					
*Backward selected model*										
Cooperativeness	0.24	0.17	0.08	2.20	**0.031**	5.72	3, 71	**0.002**	0.19	−5.18
Harm avoidance	−0.24	−0.09	0.04	−2.24	**0.028**					
Reward dependence	0.29	0.18	0.07	2.64	**0.010**					
*Separate regression models*										
Novelty seeking	−0.26	−0.82	0.36	−2.27	**0.026**	5.15	1, 73	**0.026**	0.07	3.42***
Harm avoidance	−0.18	−0.53	0.34	−1.54	0.128	2.37	1, 73	0.128	0.03	3.38***
Reward dependence	0.31	0.92	0.33	2.80	**0.006**	7.85	1, 73	**0.007**	0.10	3.38***
Persistence	0.15	0.47	0.36	1.31	0.194	1.72	1, 73	0.194	0.02	3.39***
Self-directedness	0.21	0.64	0.35	1.82	0.073	3.32	1, 73	0.073	0.04	3.39***
Cooperativeness	0.26	0.79	0.35	2.28	**0.026**	5.18	1, 73	**0.026**	0.07	3.39***
Self-transcendence	0.20	0.61	0.35	1.74	0.085	3.04	1, 73	0.085	0.04	3.33***

Note. *P* values < .05 are displayed in bold. ***p < .001

### Research question 2: do childhood temperament and character features predict mother–adolescent dyadic interaction quality above and beyond mental health variables?

When additionally to reward dependence, harm avoidance, and cooperativeness, child and maternal mental health variables and early interactional dysfunction were entered, the overall model was significant (*R*^2^ = 0.30, *F*(10, 64) = 3.03, *p* = 0.004). In this multiple regression, reward dependence (*β* = 0.29, *p* = 0.015) and current maternal stress negatively (*β* = −0.30, *p* = 0.031) predicted dyadic interaction quality at age 14. Starting the backward selection process from here, the model predicting dyadic interaction quality with the best fit included reward dependence at age 5 (*β* = 0.34, *p* = 0.002), interactional dysfunction at age 5 (*β* =−0.27, *p* = 0.011) and current maternal stress (*β* = −0.23, *p* = 0.031). The model significantly explained 25% of variance in dyadic interaction quality (*R*^2^ = 0.25, *F*(3, 70) = 7.65, *p* < 0.001) (for all models including temperament and character plus covariates, see Supplement Table S2). Additional separate regressions of those predictors making up the final model show that most variance in this model is explained by reward dependence *(R*^2^ = 0.10, see Table 1) and interactional dysfunction (*R*^2^ = 0.10, *F*(1, 72) = 8.38, *β* = −0.32, *p* = 0.005), and only little variance in dyadic interaction is attributable to current maternal stress, which on its own was not a significant predictor of mother–adolescent interaction (*R*^2^ = 0.04, *F*(1, 74) = 2.78, *β* = −0.19, *p* = 0.110.)

Next, multiple regression analyses were performed with the separate temperament and character scales to investigate if their respective effects were differentiable from those of selected covariates. Novelty seeking significantly predicted dyadic interaction above the effect of maternal stress at age 14 (*β* = −0.26, *p* = 0.022). Novelty seeking also still held significant estimates when controlling the model for child/adolescent psychopathology (age 5 or 14), or maternal psychopathology (age 5 or 14). However, these covariates did not explain significant variance in dyadic behaviour and adding these to the model caused the overall models to become non-significant. Novelty seeking did not explain later dyadic interaction quality above and beyond the effect of earlier mother-reported parent–child dysfunctional interaction (age 5). Parent–child dysfunctional interaction at age 5 itself predicted interactional quality at age 14 (*β* = −0.26, *p* = 0.032).

Reward dependence significantly predicted later dyadic interaction quality even when child/adolescent psychopathology (age 5 or 14), maternal psychopathology (age 5 or 14), maternal stress (age 14), or parent–child dysfunctional interaction (age 5) were added. The overall regression model stayed significant in all instances. Maternal perceived stress at age 14 (*β* = −0.24, *p* = 0.029) and earlier parent–child dysfunctional interaction at age 5 (*β* = −0.29, *p* = 0.009) were significantly and negatively associated with quality of dyadic behaviour in adolescence in these models.

Cooperation significantly predicted later dyadic interaction quality above the effect of maternal perceived stress at age 14. Mirroring the pattern of the novelty seeking models, the coefficient of cooperation remained significant when controlling for child/adolescent psychopathology (age 5 or 14), or maternal psychopathology (age 5 or 14), but the overall regression models turned non-significant as none of these covariates significantly contributed to the model. The effect of cooperation did not remain significant when earlier parent–child dysfunctional was added (*β* = 0.19, *p* = 0.107). Earlier parent–child dysfunctional interaction (*β* = −0.27, *p* = 0.021) significantly predicted interactional quality at age 14 in this model.

Effects of harm avoidance, persistence, self-directedness, and self-transcendence on later dyadic interaction remained non-significant with any of the covariates added to the model (not displayed). See Supplement Table S3 for correlations between all study variables.

## Discussion

The present study investigated the longitudinal link between child temperament and mother–adolescent interactional quality while controlling for other possible predictors of dyadic interaction, especially parental and child mental health. Among the temperament dimensions, childhood novelty seeking negatively, and reward dependence and cooperativeness positively predicted later interaction quality; i.e., children who show high social responsiveness and attachment and their mothers tend to develop more optimal interaction patterns with each other, whereas childhood social detachment and a tendency towards impulsiveness and risk taking can challenge dyadic interaction. Contrary to most literature, measures of past and concurrent maternal and child/adolescent psychopathology did not significantly relate to mother–adolescent interaction.

Effects of novelty seeking and cooperativeness were, however, cancelled out by the effect of early dysfunctional interaction, which was a significant predictor of later dyadic interaction quality itself. Additionally, in distinct multiple regression models, childhood harm avoidance and concurrent perceived maternal stress emerged as negative predictors of observed interaction patterns in adolescence. 

Reward dependence at a moderate-effect size was the temperament trait with the strongest predictive value for mother–adolescent interactions 9 years later, remaining a significant predictor beyond all covariates and being included in all backward selected models. Along with reward dependence, mother–child dysfunctional interaction at age 5 with a moderate effect and current maternal stress with a small effect predicted mother–adolescent interaction quality; i.e., interaction quality was best predicted by a combination of child, maternal, and dyadic features which explained 25% of its variance. Interestingly, the childhood predictors played a larger role than concurrent maternal stress or any other of the concurrently measured variables. Toddler sociability, which is conceptually related to reward dependence and cooperativeness, has been shown to be positively related to parent’s sense of competence cross-sectionally [46], which could be a result of the experience of mutually satisfying interaction. The effect of earlier dysfunctional interaction also suggests a certain degree of continuity of mother–child relationship problems, even when assessed using different methodology.

As hypothesized, dyads showed less reciprocity during interaction when children displayed higher novelty seeking during childhood. Children who behave more impulsively and disorderly may pose more challenges on parenting, such as impulsive pre-schoolers receiving less-sensitive parenting in a cross-sectional study [47]. High novelty seeking may also interfere with optimal dyadic give-and-receive interaction processes, and this association is long term. Also, in concordance with our hypothesis, higher childhood cooperativeness predicted more optimal mother–adolescent interaction. One study showed that cooperativeness is a mediator of emotionally warm parenting between generations, thus proving a substantial resource in the construction of intrafamilial relationship quality [48]. As cooperativeness directly refers to the child’s ability to engage in mutually satisfying interactions and reflects age-appropriate character maturity, it may foster positive mother–child relationship outcomes. Controlling for dysfunctional interaction at age five cancelled out the originally small-to-moderate effect of novelty seeking and cooperativeness. Both traits at age 5 were already correlated with maternal self-reports of dysfunctional mother–child interaction at the time (Supplement Table S3). Early mother–child relationship difficulties explained overlapping proportions of variance with these dimensions, but relationship difficulties seemed to be the stronger predictor of ongoing maladaptive interactions. However, maternal and child psychopathology did not explain additional variance in multiple regressions.

The current findings indicate that child traits play an important role in shaping the mother–child relationship dynamic. In this community sample, the effect of child temperament was more central than that of past or concurrent child psychopathology. Whereas the questionnaires used in the current article to assess psychopathology are mainly concerned with problems occurring in the past weeks, a clinical interview measuring adolescent borderline personality disorder traits as present during the past 2 year period also related to the mother–child interaction patterns in this sample [49]. Together, this could suggest that enduring child traits rather than transient states have a long-term impact on mother–child dyadic interaction. This conclusion is further supported by findings from the backward selected model, in which childhood temperament and early interaction dysfunction show stronger effect sizes than concurrent maternal stress.

Unexpectedly, neither past nor current maternal psychopathology was linked to mother–adolescent interactions. Maternal psychopathology might not necessarily interfere with the mother–child relationship in a context where symptom levels are subclinical, and, as implied by a relatively high SES, resources are present. Yet, it should be noted that a lack of effect of maternal psychopathology on mother–child interaction has also been observed in high-risk families with higher symptom levels [25]. Both [25] and our study could be limited by relatively small variance in symptom levels, serving as a potential alternative explanation for the lack of effect. There might still be practical implications regarding the assumption of caregiver psychopathology being the central risk factor. The findings suggest that counsellors meeting families with interactional difficulties should not solely focus on psychopathology as the facilitator of these challenges, anticipating an improvement of symptoms will go along with improvement of the caregiver–child relationship. Instead, selective prevention may also include screenings of child characteristics which could challenge caregiver–child interactions, and apply interventions that focus on improving the caregiver–child interaction and relationship specifically. Our findings do not preclude that a combination of clinical levels of psychopathology and difficult temperament may exacerbate adverse effects on the caregiver–child relationship. Caregivers of children with an impulsive temperament or little social responsiveness may need support in shaping interactions in an adaptive, mutually satisfying manner. This is especially important as children high in impulsivity have been found to be more vulnerable to the effects of maladaptive parenting and relationship difficulties, putting them at higher risk for mental health problems [50, 51]. Without attributing fault and responsibility to any interactive partner, the finding that certain temperamental dispositions pose more challenges independently from caregiver or child mental health problems can inform and validate caregiver’s experiences. The heritability of temperament presumably also plays a role in this dynamic, and (mis)matches between child and caregiver could further impact the relationship [46, 52]. Crucially, the finding that early interactional dysfunction was one of the most recurring predictors in the established models shows that maladaptive interaction patterns can be pervasive, and it might take external support or intervention to break these patterns. Interventions educating caregivers about temperament have been showing promising results regarding improvement of outcomes, such as behaviour problems, academic achievement, and teacher–child relationships [53]. The combination of high novelty seeking with low reward dependence/cooperativeness is also suggestive of a risk pattern for the development of conduct disorder. Though focussing on the prediction of caregiver–child interaction specifically rather than child psychopathology as an outcome, current results therefore also link to invention programs such as The Incredible Years program designed to aid families with children with early onset conduct disorder [54]. Reducing harsh discipline, strengthening caregiver–child interactions, and teaching children social and problem-solving skills, similar programs might be suitable to address interactional difficulties which arise from challenging temperamental traits. Of note, the role of temperament in social interactions should not only be considered in the family context. Temperamental characteristics also predict success in peer relationships [55, 56], indicating that children could benefit from, e.g., (pre)school teachers being informed about how to facilitate social interactions and adjustment in the presence of challenging response tendencies.

The current study is based on the assumption that there are intercorrelations between temperament and the incorporated covariates. Early dysfunctional interaction related to all but one of the temperament scales in the way it was hypothesized for the longitudinal association with mother–adolescent interaction patterns. Maternal mental health was not related to child temperament, suggesting that there was little reason to suspect that the temperament measure was biased. Child mental health was only related to temperament when measured concurrently. There were some intercorrelations between the JTCI scales themselves, especially the character scales cooperativeness und self-directedness were associated with the temperament dimensions in ways which would support the theory that certain temperamental dispositions are beneficial to mature character development. Whereas intercorrelations between predictors can challenge the interpretability of multiple regression models, results from these models show especially those predictors which were not intercorrelated (and thus, had no overlapping effects) remained significant in the final models.

Contrary to our hypotheses, persistence, self-direction, and self-transcendence did not predict later interaction, indicating a minor role for the development of the mother–child relationship longitudinally. The role of harm avoidance was inconclusive, as separate and multiple regression led to differing results. A meta-analysis found that the temperamental trait of negative emotionality, despite an overall negative relationship with parenting, actually has an inverse relationship and is associated with more supportive parenting in samples with a higher socioeconomic status [57]. Thus, the low-risk nature of our sample might have dampened the impact of harm avoidance.

### Strengths and limitations

The multi-informant approach to child temperament was a considerable strength of the current study. The way a mother perceives her child will impact both her temperament rating of the child as well as the way she interacts with them. Given the multi-informant approach, the assessment of temperament includes not only the mother’s evaluation of the child’s characteristics but also a set of different perspectives, therefore mitigating confounding between predictor and outcome. Other strengths lie in the objective observation of caregiver–child interactions as opposed to self-reports of parenting behaviour and the 9-year longitudinal design.

Still, this study is not without its limitations. There were no observed mother–child interactions at age 5, and the parent–child dysfunctional interaction scale is only a subscale of the parenting stress inventory. Parent–child interaction at age 5 therefore was not measured objectively and more rudimentarily than dyadic interaction assessed using a video-coding scheme like the CIB at age 14. Still, the finding that there are moderate correlations between the self-report and the observed mother–child interaction 9 years later validates its use. An assessment of both temperament and caregiver–child interaction at multiple time points would be able to better inform us about the mutual influences and possible sensitive developmental periods and could be approached in future studies. Given the relatively small sample size, correction for multiple testing was too conservative, however, the risk for false positives should be considered as a limitation. It has to show whether results can be replicated in bigger, socio-demographically similar community samples. Finally, although fathers completed a questionnaire about children’s temperament at age 5, fathers’ sociodemographic variables or mental health were not part of the assessment, withholding the possibility to analyse whether effects of child temperament are independent of fathers’ characteristics.

## Conclusion

Whereas a majority of the literature examines caregiver–child interaction as an outcome of parental psychopathology and a predictor of child outcome, the current study showed that child temperament and character traits predict mother–child interaction quality longitudinally. Especially impulsivity and low social responsiveness might challenge the construction of reciprocal, synchronous dyadic interactions. These findings inform which dyads may be at risk for the development of maladaptive interaction cycles. Additionally, early maladaptive interaction in itself is a predictor of lower interaction quality in adolescence, indicating the pervasiveness of such patterns. Thus, it might be important to target caregiver–child interaction in prevention at an early stage. Selective prevention should not solely focus on psychopathology as a risk factor for the caregiver–child relationship. Counsellors should be informed about child characteristics which challenge parenting and provide guidance targeting caregiver–child interaction patterns specifically.

### Supplementary Information

Below is the link to the electronic supplementary material.

Supplementary file1 (DOCX 48 KB)

## Data Availability

Data are available from the corresponding author upon reasonable request.
