# Supplementary material for: Child temperament as a longitudinal predictor of mother–adolescent interaction quality: are effects independent of child and maternal mental health?
Source: Eur Child Adolesc Psychiatry. 2024 Jan 19;33(8):2791–801. doi: 10.1007/s00787-023-02359-6 (PMC11272696; doi:10.1007/s00787-023-02359-6)
Supplement: Supplementary file 1 — Supplementary file1 (DOCX 48 KB) [file 787_2023_2359_MOESM1_ESM.docx]

**Supplement**

**Child Temperament as a Longitudinal Predictor of Mother-Adolescent Interaction Quality: Are Effects Independent of Child and Maternal Mental Health?**

***European Child and Adolescent Psychiatry***

Leonie Fleck, Anna Fuchs, Katharina Williams, Eva Moehler, Franz Resch, Julian Koenig, Michael Kaess

**Corresponding author**: Michael Kaess, University Hospital of Child and Adolescent Psychiatry and Psychotherapy, University of Bern, Stöckli, Bolligenstrasse 141c, 3000 Bern 60, Switzerland; Email: [michael.kaess@upd.ch](mailto:michael.kaess@upd.ch)

**Table S1**

*Descriptives of study variables*

| Variable | N | Mean | Std. Dev. | Min | Max |
| --- | --- | --- | --- | --- | --- |
| (1) dyadic interaction quality t6 | 76 | 3.274 | 3.14 | -6.08 | 7.83 |
| (2) novelty seeking t5 | 75 | 19.13 | 6.25 | 7.33 | 35.67 |
| (3) harm avoidance t5 | 75 | 17.84 | 8.65 | 1 | 48 |
| (4) reward dependence t5 | 75 | 28.44 | 4.80 | 19.33 | 43 |
| (5) persistence t5 | 75 | 31.02 | 5.47 | 17 | 43.67 |
| (6) self-directedness t5 | 75 | 34.10 | 4.60 | 19.33 | 42.33 |
| (7) cooperativeness t5 | 75 | 28.76 | 4.40 | 18.33 | 38 |
| (8) self-transcendence t5 | 75 | 21.87 | 4.90 | 11 | 32.50 |
| (9) child mental health problems (SDQ) t5 | 74 | 5.23 | 3.02 | 0 | 15 |
| (10) dysfunctional interaction (PCDI) t5 | 74 | 17.60 | 4.16 | 12 | 30 |
| (11) maternal psychopathology (SCL) t5 | 75 | .18 | .20 | 0 | 1.20 |
| (12) child mental health problems (SDQ) t6 | 76 | 9.03 | 5.218 | 0 | 23 |
| (13) maternal psychopathology (BSI) t6 | 76 | .27 | .22 | 0 | 1.22 |
| (14) maternal stress (PSQ) t6 | 76 | 33.716 | 16.46 | 3.33 | 78.33 |
| *Note.* SDQ = Strengths and Difficulties Questionnaire. PCDI = Child Dysfunctional Interaction. SCL-90R = Symptom Checklist. BSI = Brief Symptom Inventory. PSQ = Perceived Stress Questionnaire. t5 = child age 5. t6 = child age 14. | | | | | |

**Table S2**

*Multiple Regression Analyses: Prediction of Dyadic Interaction Quality by Temperament, Character and Psychosocial Covariates.*

|  |  | *Full regression model* | | | | | | | | | |  |
| --- | --- | --- | --- | --- | --- | --- | --- | --- | --- | --- | --- | --- |
| Variables | | | *β* | *b* | *SE* | *t* | *p* | F | df | *p(F)* | *R²* | *intercept* |
| Reward dependence t5 | | | .30 | .92 | .35 | 2.62 | **.011** | 3.03 | 10,64 | **.004** | .31 | 3.49*** |
| Harm avoidance t5 | | | -.19 | -.57 | .42 | -1.35 | .183 |  |  |  |  |  |
| Cooperativeness t5 | | | .27 | .83 | .43 | 1.94 | **.057** |  |  |  |  |  |
| Child mental health problems (SDQ) t5 | | | .15 | .46 | .45 | 1.03 | .208 |  |  |  |  |  |
| Child mental health problems (SDQ) t6 | | | -.02 | -.05 | .35 | -.15 | .477 |  |  |  |  |  |
| Maternal psychopathology (SCL-90R) t5 | | | .03 | .09 | .37 | .24 | .885 |  |  |  |  |  |
| Maternal psychopathology (BSI-18) t6 | | | .08 | .25 | .43 | .58 | .611 |  |  |  |  |  |
| Maternal stress (PSQ) t6 | | | -.30 | -.91 | .42 | -2.19 | **.031** |  |  |  |  |  |
| Dysfunctional interaction (PCDI) t5 | | | -.17 | -.51 | .39 | -1.32 | .191 |  |  |  |  |  |
|  |  | *Backward selected model* | | | | | | | | | |  |
| Reward dependence t5 | | | .34 | 1.03 | .32 | 3.19 | **.002** | 7.65 | 3, 70 | **<.001** | .25 | 3.49 |
| Dysfunctional interaction (PCDI) t5 | | | -.27 | -.82 | .31 | -2.62 | **.011** |  |  |  |  |  |
| Maternal stress (PSQ) t6 | | | -.23 | -.71 | .32 | -.2.21 | **.031** |  |  |  |  |  |
|  |  | *Separate regressions* | | | | | | | | | |  |
| Novelty seeking t5 | | | -.29 | -.94 | .45 | -2.10 | **.039** | 2.59 | 2, 71 | .082 | .07 | 3.42*** |
| Child mental health problems (SDQ) t5 | | | .07 | .21 | .44 | .49 | .627 |  |  |  |  |  |
| Novelty seeking | | | -.25 | -.79 | .36 | -2.16 | **.034** | 2.77 | 2, 72 | .069 | .07 | 3.42*** |
| Child mental health problems (SDQ) t6 | | | -.08 | -.23 | .35 | -.66 | .509 |  |  |  |  |  |
| Novelty seeking t5 | | | -.26 | -.84 | .37 | -2.29 | **.025** | 2.62 | 2, 72 | .079 | .07 | 3.43*** |
| Maternal psychopathology (SCL-90R) t5 | | | -.05 | -.14 | .34 | -.40 | .689 |  |  |  |  |  |
| Novelty seeking t5 | | | -.26 | -.85 | .36 | -2.33 | **.023** | 2.97 | 2, 72 | .057 | .08 | 3.42*** |
| Maternal psychopathology (BSI-18) t6 | | | -.10 | -.31 | .35 | -.90 | .369 |  |  |  |  |  |
| Novelty seeking t5 | | | -.26 | -.83 | .36 | -2.35 | **.022** | 4.30 | 2, 72 | **.017** | .11 |  |
| Maternal stress (PSQ) t6 | | | -.20 | -.62 | .34 | -1.82 | .073 |  |  |  |  |  |
| Novelty seeking t5 | | | -.15 | -.49 | .39 | -1.27 | .208 | 5.03 | 2, 71 | **.009** | .12 | 3.46*** |
| Dysfunctional interaction (PCDI) t5 | | | -.26 | -.79 | .36 | -2.19 | **.032** |  |  |  |  |  |
| Reward dependence t5 | | | .36 | 1.10 | .34 | 3.22 | **.002** | 5.62 | 2, 71 | **.005** | .14 | 3.46*** |
| Child mental health problems (SDQ) t5 | | | -.16 | -.49 | .35 | -1.40 | .165 |  |  |  |  |  |
| Reward dependence t5 | | | .30 | .89 | .33 | 2.71 | **.008** | 4.13 | 2, 72 | **.020** | .10 | 3.37*** |
| Child mental health problems (SDQ) t6 | | | -.08 | -.23 | .34 | -.68 | .500 |  |  |  |  |  |
| Reward dependence t5 | | | .32 | .94 | .33 | 2.83 | **.006** | 4.00 | 2, 72 | **.023** | .10 | 3.39*** |
| Maternal psychopathology (SCL-90R) t5 | | | -.05 | -.16 | .34 | -.49 | .629 |  |  |  |  |  |
| Reward dependence t5 | | | .31 | .92 | .33 | 2.81 | **.006** | 4.21 | 2, 72 | **.019** | .10 | 3.37*** |
| Maternal psychopathology (BSI-18) t6 | | | -.09 | -.27 | .34 | -.78 | .438 |  |  |  |  |  |
| Reward dependence t5 | | | .34 | 1.01 | .32 | 3.15 | **.002** | 6.62 | 2, 72 | **.002** | .16 | 3.38*** |
| Maternal stress (PSQ) t6 | | | -.24 | -.74 | .33 | -2.23 | **.029** |  |  |  |  |  |
| Reward dependence t5 | | | .30 | .93 | .33 | 2.82 | **.006** | 8.57 | 2, 71 | **.001** | .19 | 3.48*** |
| Dysfunctional interaction (PCDI) t5 | | | -.29 | -.85 | .32 | -2.69 | **.009** |  |  |  |  |  |
| Cooperativeness t5 | | | .27 | .82 | .37 | 2.12 | **.037** | 2.65 | 2, 71 | .078 | .07 | 3.41*** |
| Child mental health problems (SDQ) t5 | | | .01 | .03 | .44 | .07 | .942 |  |  |  |  |  |
| Cooperativeness t5 | | | .25 | .76 | .35 | 2.15 | **.035** | 2.74 | 2, 72 | .071 | .07 | 3.38*** |
| Child mental health problems (SDQ) t6 | | | -.07 | -.21 | .35 | -.59 | .554 |  |  |  |  |  |
| Cooperativeness t5 | | | .26 | .81 | .35 | 2.30 | **.025** | 2.64 | 2, 72 | .078 | .07 | 3.40*** |
| Maternal psychopathology (SCL-90R) t5 | | | -.05 | -.14 | .34 | -.40 | .694 |  |  |  |  |  |
| Cooperativeness t5 | | | .27 | .83 | .35 | 2.39 | **.020** | 3.10 | 2, 72 | .051 | .08 | 3.39*** |
| Maternal psychopathology (BSI-18) t6 | | | -.12 | -.35 | .35 | -1.01 | .315 |  |  |  |  |  |
| Cooperativeness t5 | | | .28 | .85 | .34 | 2.49 | **.015** | 4.67 | 2, 72 | **.012** | .11 | 3.39*** |
| Maternal stress (PSQ) t6 | | | -.22 | -.67 | .34 | -1.99 | .051 |  |  |  |  |  |
| Cooperativeness t5 | | | .19 | .58 | .35 | 1.63 | .090 | 5.62 | 2, 71 | **.005** | .14 | 3.46*** |
| Dysfunctional interaction (PCDI) t5 | | | -.27 | -.81 | .34 | -2.35 | **.021** |  |  |  |  |  |

*Note*. *P* values <.05 are displayed in bold. SDQ = Strengths and Difficulties Questionnaire. PCDI = Child Dysfunctional Interaction. SCL-90R = Symptom Checklist. BSI = Brief Symptom Inventory. PSQ = Perceived Stress Questionnaire. t5 = child age 5. t6 = child age 14.

**Table S3**

*Correlations of all study variables*

| Variables | (1) | (2) | (3) | (4) | (5) | (6) | (7) | (8) | (9) | (10) | (11) | (12) | (13) |
| --- | --- | --- | --- | --- | --- | --- | --- | --- | --- | --- | --- | --- | --- |
| (1) dyadic mother-adolescent interaction quality t6 |  |  |  |  |  |  |  |  |  |  |  |  |  |
|  |  |  |  |  |  |  |  |  |  |  |  |  |  |
| (2) novelty seeking t5 | **-.257** |  |  |  |  |  |  |  |  |  |  |  |  |
|  | **(.026)** |  |  |  |  |  |  |  |  |  |  |  |  |
| (3) harm avoidance t5 | -.177 | .160 |  |  |  |  |  |  |  |  |  |  |  |
|  | (.128) | (.139) |  |  |  |  |  |  |  |  |  |  |  |
| (4) reward dependence t5 | **.312** | -.097 | .053 |  |  |  |  |  |  |  |  |  |  |
|  | **(.006)** | (.369) | (.623) |  |  |  |  |  |  |  |  |  |  |
| (5) persistence t5 | .152 | **-.441** | -.181 | .113 |  |  |  |  |  |  |  |  |  |
|  | (.194) | **(.000)** | (.094) | (.297) |  |  |  |  |  |  |  |  |  |
| (6) self-directedness t5 | .209 | **-.374** | **-.710** | -.085 | **.567** |  |  |  |  |  |  |  |  |
|  | (.073) | **(.000)** | **(.000)** | (.434) | **(.000)** |  |  |  |  |  |  |  |  |
| (7) cooperativeness t5 | **.257** | **-.790** | .068 | .197 | **.264** | .149 |  |  |  |  |  |  |  |
|  | **(.026)** | **(.000)** | (.531) | (.068) | **(.014)** | (.169) |  |  |  |  |  |  |  |
| (8) self-transcendence t5 | .200 | .106 | .128 | **.457** | .099 | .056 | -.036 |  |  |  |  |  |  |
|  | (.085) | (.329) | (.239) | **(.000)** | (.360) | (.607) | (.741) |  |  |  |  |  |  |
| (9) child mental health problems (SDQ) t5 | -.101 | **.619** | **.446** | .098 | **-.502** | **-.576** | **-.450** | .084 |  |  |  |  |  |
|  | (.391) | **(.000)** | **(.000)** | (.374) | **(.000)** | **(.000)** | **(.000)** | (.444) |  |  |  |  |  |
| (10) dysfunctional mother-child interaction (PCDI) t5 | **-.323** | **.406** | **.449** | -.131 | **-.303** | **-.493** | **-.304** | -.044 | **.396** |  |  |  |  |
|  | **(.005)** | **(.000)** | **(.000)** | (.231) | **(.005)** | **(.000)** | **(.005)** | (.687) | **(.000)** |  |  |  |  |
| (11) maternal psychopathology (SCL) t5 | -.004 | -.085 | .084 | .161 | .043 | -.177 | .091 | .084 | .008 | .103 |  |  |  |
|  | (.973) | (.433) | (.441) | (.138) | (.691) | (.101) | (.402) | (.440) | (.939) | (.350) |  |  |  |
| (12) child mental health problems (SDQ) t6 | -.124 | .126 | -.073 | -.102 | -.007 | .124 | -.156 | .085 | .135 | -.001 | .162 |  |  |
|  | (.285) | (.283) | (.533) | (.385) | (.953) | (.291) | (.181) | (.467) | (.251) | (.991) | (.165) |  |  |
| (13) maternal psychopathology (BSI) t6 | -.081 | -.081 | .041 | .018 | .116 | .020 | .125 | .021 | -.071 | -.034 | **.468** | .207 |  |
|  | (.489) | (.492) | (.725) | (.876) | (.322) | (.867) | (.286) | (.858) | (.548) | (.776) | **(.000)** | (.072) |  |
| (14) maternal stress (PSQ) t6 | -.190 | -.023 | .131 | .137 | .214 | -.047 | .090 | .011 | .113 | .042 | **.406** | **.287** | **.559** |
|  | (.100) | (.842) | (.262) | (.242) | (.065) | (.687) | (.443) | (.927) | (.336) | (.723) | **(.000)** | **(.012)** | **(.000)** |

*Note.* SDQ = Strengths and Difficulties Questionnaire. PCDI = Parent-Child Dysfunctional Interaction. SCL = Symptom Checklist. BSI = Brief Symptom Inventory. PSQ = Perceived Stress Scale. t5 = child age 5. t6 = child age 14. Values in brackets present *p*-values. Significant associations (p<.05) are presented in bold.
